# Direction matters: Comparing post-editing and human translation effort and quality

**DOI:** 10.1371/journal.pone.0328511

**Published:** 2025-07-29

**Authors:** Sanjun Sun, Hao Wang, Yanfang Jia

**Affiliations:** 1 School of English and International Studies, Beijing Foreign Studies University, Beijing, China; 2 Translation and Interpreting Studies Department, Hunan Normal University, Changsha, China; Woldia University, ETHIOPIA

## Abstract

This study investigates the critical yet understudied influence of translation direction on both neural machine translation post-editing (PE) and human translation (HT) processes. While previous research has compared PE and HT, the interaction between translation mode and directionality—particularly for linguistically distant languages—remains poorly understood. We address this gap by examining how L1-L2 (Chinese-English) versus L2-L1 (English-Chinese) directionality affects translation performance across multiple dimensions. Thirty-one native Chinese translators completed both PE and HT tasks in both language directions, with their processes documented through keylogging and screen recording. Our findings reveal that PE generally outperforms HT in speed, technical efficiency, and output quality, but these advantages manifest differently depending on translation direction. The PE advantage was significantly more pronounced in the L2-L1 direction. Conversely, perceived task difficulty was primarily determined by directionality rather than translation mode, with L1-L2 translation consistently rated more challenging regardless of whether performed through PE or HT. These results demonstrate the complex interplay between translation mode and directionality, challenging the simplistic view that PE universally reduces effort compared to HT. Our findings have important implications for translator training, workflow optimization, and fair compensation models that account for both translation mode and direction.

## 1. Introduction

Machine translation post-editing (PE) has become an integral part of contemporary translation workflows, allowing translators to efficiently edit raw machine-generated output [1,2]. The rapid advancement of neural machine translation (NMT) technology has further accelerated the adoption of PE in professional translation settings. As the industry evolves, understanding the nuances between PE and conventional human translation (HT) across different language pairs and directions becomes crucial for optimizing workflows and training programs.

Despite growing research in this field, gaps remain in fully understanding how PE compares to conventional human translation [3], especially regarding the role of translation directionality [4] (i.e., translation into the translator’s first/native language, L1, versus their second language, L2) in the NMT scenario. While our previous work investigated the interaction of source text complexity and MT quality on translation difficulty [5], this study specifically isolates the impact of translation directionality on effort, quality, and perceived difficulty in NMT PE versus HT. Accordingly, this study focuses on the Chinese-English language pair, examining the complex interplay between translation mode (PE vs. HT) and directionality (L1-L2 vs. L2-L1) across these dimensions.

This study addresses these gaps through an in-depth comparative analysis of PE and human translation in the Chinese-English language pair. Building on Krings’ [6] framework of translation as a multifaceted cognitive activity, we examine temporal effort, technical effort, output quality, and perceived difficulty. Our robust experimental design incorporates keylogging, screen recording, and questionnaire data to answer two main research questions:

(1) How does PE compare to human translation in terms of temporal effort, technical effort, product quality, and perceived difficulty?
(2) How does translation direction (from L1 to L2 vs. from L2 to L1) impact these factors for both PE and human translation?

This research contributes to ongoing discussions about the evolving relationship between machine and human roles in translation, potentially refining existing theoretical models. By focusing on the Chinese-English pair, we provide empirical insights that can inform translation pedagogy, workflow optimization, and future interdisciplinary research at the intersection of translation studies, linguistics, and machine learning.

## 2. Comparisons between post-editing and human translation

In the context of PE and HT, quite a few studies have explored the similarities and differences between these two translation modes. These comparative analyses have provided valuable insights into various aspects such as effort expenditure, output quality, and the role of translation direction. This section presents a literature review that examines previous research on the comparisons between PE and HT.

### 2.1. Effort expenditure: Time and technical aspects

Understanding the effort involved in translation tasks is crucial for comparing PE and HT processes. Krings [6] classified translation effort into temporal, technical, and cognitive categories, providing a framework for analyzing the multifaceted nature of translation work.

Recent large-scale research by Terribile [7], analyzing data from 90 million words translated across 11 language pairs, found that PE was on average 66% faster than HT at the translation stage. However, this advantage varied dramatically between language pairs, ranging from a 130% speed increase to a 7% decrease. Interestingly, PE revision was also found to be 38% faster than HT revision on average, despite similar quality expectations.

These findings align with earlier studies across various language pairs. For the Chinese-English language pair, Sun et al. [8] conducted a comprehensive eye-tracking study comparing PE and HT. They found that PE tasks were generally completed faster and with less cognitive effort than HT tasks, even for this linguistically distant language pair. Their study also revealed that the advantage of PE over HT in terms of speed and technical effort was more pronounced for more educated translators and for less technical texts.

The relationship between effort and output in PE is complex and can vary based on multiple factors. Cui, et al. [9] indicated that PE generally demands less effort and enhances productivity across multiple text types, except for legal texts. Wang & Daghigh [10] emphasized the significant influence of text type on a translator’s time investment, mental process, and technical input in both HT and PE processes.

Jia & Sun [5] demonstrated that PE was significantly less difficult than HT only when high-quality MT was paired with complex texts. Koponen [2] underscored that while post-editing high-quality MT can increase productivity, editing poor MT can be unproductive. Yamada [11] observed that students displayed a lower error correction rate in post-editing neural MT due to its human-like errors, highlighting the complexity of PE.

Terribile’s [7] research found only moderate to weak correlations between PE speed and edit distance, a measure of technical effort. This aligns with earlier findings by Krings [6] and Moorkens et al. [12] that cognitive effort is not always strongly correlated with temporal and technical effort. Macken et al. [13] noted that while MT generally leads to productivity gains, the connection between technical and temporal effort was weak.

Various methods such as think-aloud protocols, keylogging, and eye-tracking have been used to explore cognitive effort [14,15]. Vieira [16] emphasized the importance of understanding cognitive effort in translation processes, highlighting the intricate relationship between effort, speed, and quality.

As translation technologies continue to evolve, ongoing research will be crucial to fully understand the changing dynamics of effort in PE versus HT across different language pairs and translation contexts.

### 2.2. Evaluating output: Quality in post-editing vs. human translation

The quality of output in PE versus HT has been a topic of ongoing discussion in the translation field. Studies have examined this aspect from various perspectives, often yielding context-dependent results.

Fiederer and O’Brien [17] compared the quality of HT, machine translation, and post-edited machine translation in terms of clarity, accuracy, and style. They found that post-edited translations were rated as clearer and more accurate than human translations, though human translations were considered to have better style. This suggests that PE can produce high-quality output in certain aspects, while still lacking in others.

Garcia [18] provided further evidence supporting the potential of PE to produce higher quality translations. In a study involving translation students, Garcia found that post-edited translations received better quality scores than those translated from scratch, regardless of language direction, text difficulty, or translator performance level. This quality advantage was particularly pronounced when participants were translating into their second language.

However, the quality of PE output can vary depending on the specific context. Cui et al. [9] found that while PE generally enhances translation quality, there was variation in effort and potentially quality when dealing with legal texts. This suggests that the quality of PE may not consistently surpass that of HT in all domains.

Recent research by Sun et al. [8] provides valuable insights into the Chinese-English language pair in the context of PE and HT. Their study compared the quality of human translations, post-edited versions, and machine translations for Chinese-to-English tasks. They found no significant differences in quality ratings between human translations and post-edited versions, regardless of the translator’s education level (undergraduate or postgraduate) or the text domain (general, political, or economic). However, both human translations and post-edited versions significantly outperformed raw machine translations. This suggests that for Chinese-to-English translation, PE can achieve comparable quality to HT while potentially offering productivity gains.

The MT system used can also impact PE quality. Yamada [11] compared student PE potential using different MT systems and found that while NMT produced fewer errors, it did not necessarily enable students to meet professional translation standards. The NMT outputs often contained human-like errors, making it challenging for student translators to post-edit.

Recent research by Daems et al. [19] and Daems & Macken [20] supports the notion that PE and HT are becoming more similar. They highlighted that the assumption that PE and HT revision are distinct may not hold true anymore with the advent of NMT systems.

Considering these studies, it becomes evident that the comparison of quality between post-edited machine translations and human translations is context-specific. Factors such as text type, MT system, translator proficiency, language pair, and translation direction can all influence the outcome. While some studies suggest PE can produce translations of comparable or higher quality than HT, especially for non-professional translators or when working into L2, the results may vary across different scenarios and language pairs. The findings from Sun et al. [8] for the Chinese-English pair contribute to this nuanced understanding, suggesting that PE can achieve quality parity with HT in this language direction.

### 2.3. The role of directionality in post-editing and human translation

Directionality in translation tasks has been a subject of interest in translation studies, with researchers exploring the differences between direct translation (L2 to L1) and inverse translation (L1 to L2). While studies have indicated cognitive effort variances between direct and inverse translation in HT using eye-tracking techniques [21], the impact of directionality in PE for the Chinese-English language pair requires further investigation.

Research findings on the influence of directionality in PE have been mixed. Toledo Báez [22] compared PE into L1 and L2 in terms of quality and speed, finding that PE into L1 generally yielded higher quality and faster results, although the differences were largely not statistically significant. Similarly, Da Silva et al. [4] reported that directionality did not significantly influence total task time. However, other studies suggest contrary findings, with participants taking longer and employing more segmentation and recursiveness when translating into the L2 [23,24]. These conflicting results highlight the need for further investigation into the role of directionality in both PE and HT.

Fung [25] examined the implications of directionality in PE and argued that the mother-tongue principle, which suggests translators should work into their native language, is often challenged due to a lack of expertise in certain language directions. The study explored whether native Chinese translators performed differently from native English translators when post-editing a machine-translated patent text from Chinese into English. The findings indicated that L1 post-editors did not necessarily outperform their L2 counterparts, suggesting that directionality does not automatically determine PE performance. These findings raise questions about the interaction between directionality and translator expertise in different language pairs and text types.

Stasimioti et al. [26] investigated the role of directionality in PE, specifically in the English-Greek language pair. Their study compared the cognitive, temporal, and technical effort expended by translators in fully post-editing NMT output in L1 (Greek) and L2 (English). The results revealed that inverse PE (L1-L2) required less time, fewer keystrokes, and imposed a lower cognitive load on translators compared to direct PE (L2-L1). Importantly, the research indicated that directionality did not imply differences in translation quality.

The existing literature underscores the complex role of directionality in both PE and HT. While it influences various aspects of the translation process, its effects appear to be context-dependent, varying across language pairs, text types, and individual translator characteristics. Moreover, the interaction between directionality and translation mode (PE vs. HT) remains underexplored, particularly in the Chinese-English language pair. Further research is needed to understand how directionality affects effort, quality, and perceived difficulty in both PE and HT across different language combinations and text types.

## 3. Method

### 3.1. Participants

The study involved 31 students from Beijing Foreign Studies University, all specializing in English-Chinese translation and interpreting. This group was comprised of 16 third-year undergraduate students (average age 21.5) and 15 first-year graduate students (average age 23.9). All the participants were native speakers of Mandarin Chinese and had undergone conventional translation training, encompassing both theoretical and practical aspects. While formal English proficiency testing was not conducted for this study, based on the university’s admission standards and the participants’ academic performance, their English proficiency was estimated to be equivalent to an IELTS score of 7.0 or above. This level indicates a high degree of operational command of the English language.

Participation in the study was completely voluntary. Each participant gave their consent for the collection of their identifiable information for potential future communication, while being assured that it would not be used for any other purposes. As compensation for their time and effort, each participant received 130 yuan (approximately USD 20).

### 3.2. Source texts and machine translation selection

Two short informative passages, one in English (129 words) and one in Chinese (205 characters), were selected from technology-related news articles. The word count ratio between the English and Chinese passages was roughly 3:5, which aligns with the typical ratio observed in corresponding texts between these two languages.

In choosing the source texts, consideration was given to their equivalent translation difficulty level, which was based on three translation instructors’ subjective evaluation, and the “rich points” concept [27], which pertains to potential difficulties that could arise in linguistic, textual, or extralinguistic domains, among others. The instructors, all with PhDs in translation studies and over 10 years of professional translation experience, were selected for their expertise in both languages and familiarity with translation assessment.

The neural machine translations for the selected texts were directly obtained from Google Translate, chosen for its status as one of the leading MT engines available. Google Translate is known for its continuous improvements in translation quality, particularly for widely used language pairs such as Chinese-English. The same three translation instructors evaluated their quality using the adequacy and fluency criteria from TAUS’s Dynamic Quality Evaluation Framework [28]. The evaluation was done on a 4-point Likert scale: For adequacy: A score of “1” represents none of the meaning in the source text contained in the target text, while a score of “4” represents all of the meaning preserved in the target text; For fluency: A score of “1” indicated incomprehensibility, while a score of “4” indicated flawless fluency.

The L2-L1 (English to Chinese) machine translation had a mean adequacy score of 3.70 and a mean fluency score of 3.53, while the L1-L2 (Chinese to English) translation had a mean adequacy score of 3.80 and a mean fluency score of 3.35. Overall, the machine translations for the selected texts demonstrated consistently high quality. This is noteworthy because the quality of machine translation plays a crucial role in the comparison between PE and HT.

### 3.3. Data collection

To capture the multiple dimensions of translation effort as outlined by Krings [6], we adopted a triangulation approach incorporating three data collection techniques: keylogging, screen recording, and post-translation questionnaires. This approach allowed us to measure temporal effort (through task duration), technical effort (through keylogging data), and cognitive effort (through perceived difficulty ratings).

Keylogging, facilitated through the software Translog-II [29], was utilized to track keyboard activities, specifically logging text modification events (insertions and deletions) and their associated timestamps, throughout the translation process. The software produces detailed logfiles and summary reports containing user activity data. These logs and reports allow for the direct extraction and quantification of key technical effort variables, such as the counts of specific insertion and deletion actions, and the calculation of time durations for tasks or segments [30]. These data can be further analyzed using external tools such as the TPR-DB (Translation Process Research Database [30]).

Screen recording was performed using EVCapture (www.ieway.cn) to visually document the translation process. Although not conducive to rapid quantitative analysis, screen recordings offered a tangible and easily traceable record of the process, thereby aiding in the interpretation of the quantitative data.

Post-translation questionnaires were used to gauge participants’ perceived difficulty of the translation task. These questionnaires employed a modified version of the NASA Task Load Index (NASA-TLX) specifically adapted for the assessment of translation task difficulty [31]. This was administered immediately following each task to ensure its validity and accuracy. The customized NASA-TLX consisted of four workload-related subscales: Mental Demand, Effort, Frustration Level, and Performance, each of which was rated on a scale from 1 to 10. The translation difficulty score was derived from the average of the scores on these four subscales, with an adjustment made for the Performance subscale, where the score was subtracted from 10.

### 3.4. Experiment procedures

This experiment has been approved by the Research Ethical Review Board at Beijing Foreign Studies University on November 15^th^ 2023 (Ref No: ETH2024111501). A written consent form was obtained from each participant before the experiment. Data were collected from November 20, 2023 to February 25, 2024. The experimental process followed a within-subjects design, consisting of two distinct phases separated by a two-month interval. In each phase, participants randomly performed both L2-L1 (English-Chinese) translation and L1-L2 (Chinese-English) translation tasks. The first phase focused on HT tasks, while the second phase involved PE tasks. Importantly, the same source texts were used in both phases, ensuring consistency across the experimental conditions.

Before embarking on the tasks, participants were briefed about the procedure, signed the Informed Consent Form, and provided basic personal information. The experiment commenced with the EVCapture program’s activation to record the screen. The participants then initiated the translation task and keylogging session via the Translog-II User Interface. Upon completion, the recorded keystroke activities were saved, and the screen recording session ended. Directly after, participants appraised the task difficulty via the adapted NASA-TLX scales.

To mitigate the potential influence of carryover effects and ensure the independence of the experiment’s two phases, participants were intentionally kept unaware of the details regarding the second phase. Instead, they were informed in advance about the existence of two upcoming translation tasks. This approach aimed to prevent participants from deliberately memorizing their translations from the initial phase.

In order to ensure thorough and precise documentation of all activities, participants were required to solely rely on computers for their translation tasks. They were permitted to utilize online resources as references during the translation process. It is crucial to emphasize that participants received explicit instructions not to consult machine translation at any point. Even in the case of PE tasks, they were only allowed to work with the provided machine translation output. This precautionary measure was implemented to uphold the integrity of the HT tasks and prevent any potential influence or bias introduced by external machine translation resources.

Upon experiment completion, a compilation of data was accumulated from 31 participants, including 124 logfiles, 124 translation task difficulty evaluation scales, and 124 screen recordings. Following this, the three translation instructors graded the 124 target texts. Upon mutual agreement of a rating criterion, each instructor independently graded all texts, utilizing a 0–100 score range, with higher scores signifying superior quality. Inter-rater reliability was validated, and the definitive quality score for each text was calculated as the mean value of the three scores.

The collected data, composed of five numerical variable sets (duration, count of deletions, count of insertions, quality scores, and perceived difficulty scores) and three categorical variables (translation mode, direction, education level), was systematically catalogued and preserved in a.csv format, allowing for easy access and analysis.

### 3.5. Data processing

To accurately interpret the results and guide the selection of the most appropriate statistical methods, it was essential to evaluate the distribution of these variables. The Shapiro-Wilk test, a normality test, was employed. In instances where the *p*-value was less than 0.05, the null hypothesis that the data follow a normal distribution was rejected. The variables “Duration”, “Count of deletions”, and “Count of insertions” did not follow a normal distribution, necessitating alternative methods of analysis.

For the variable “Duration”, a generalized linear mixed model (GLMM) with a Gamma distribution and log link function was used. This decision was based on the fact that “Duration” is a continuous variable and might follow a Gamma distribution, a common distribution for positive-valued continuous variables.

On the other hand, the variables “Count of deletions” and “Count of insertions” are count data and were likely to follow a Poisson or negative binomial distribution. Thus, a GLMM with a Poisson distribution was chosen for these types of data. The effect of translation mode and directionality on these variables was assessed, with the participant treated as a random effect.

Similarly, linear mixed-effects models were fitted to examine the effects of translation mode and directionality on product quality scores and perceived difficulty of tasks.

Prior to finalizing the quality scores of the 124 target texts, the inter-rater reliability was examined to ensure a consistent grading scale among the three participating translation instructors. The Intraclass Correlation Coefficient (ICC) was utilized to assess the level of agreement among the raters, which revealed a significant level of agreement, ICC(2, 3) = 0.753, p < .001. This outcome validated the decision to compute the final quality score for each text as the mean of the three raters’ scores.

## 4. Results

This section presents the findings on the impact of translation mode (PE vs. HT) and directionality (L1-L2 vs. L2-L1) on four key aspects of the translation process: temporal effort, technical effort, output quality, and perceived task difficulty. These analyses directly address our two main research questions regarding the comparison between PE and HT, and the role of directionality in these processes.

### 4.1. Temporal effort in post-editing and human translation

This analysis examines the temporal effort required for PE and HT tasks across both language directions (L1-L2 and L2-L1). Specifically, we address two research questions: 1) Is there a significant difference in translation duration between HT and PE? 2) How does the direction of translation (L1-L2 vs. L2-L1) impact the duration of HT and PE tasks?

A generalized linear mixed model with a gamma distribution and a log link function was used to assess the effect of translation mode and directionality on the speed of translation, with participant as a random effect. The model was found to be significant (AIC = 3571.5, BIC = 3585.6, log-likelihood = −1780.7, df residual = 119).

**Fig 1:**
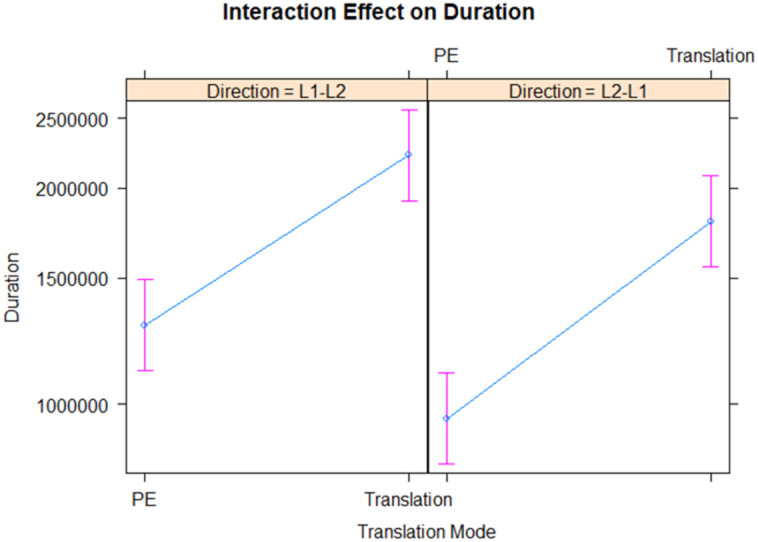
Interaction between translation mode and direction on duration.

Both translation mode (χ^2(1) = 178.83, p < .001) and direction (χ^2(1) = 34.24, p < .001) had significant effects on the duration of translation. Specifically, as shown in Fig 1, HT took longer than PE (β = 0.587, SE = 0.044, p < .001), and translations from L1-L2 were lengthier compared to those from L2-L1 (β = 0.256, SE = 0.044, p < .001). The interaction effect between translation mode and directionality was not statistically significant (χ²(1) = 1.014, p = .314), suggesting the effect of translation mode on duration is consistent across the directionality of translation.

In summary, both translation mode and direction significantly influence the duration of the translation process, with human translations and L1-L2 translations being more time-consuming. However, the influence of translation mode on duration does not significantly differ between the two directionality conditions. Regardless of whether the task is from L1 to L2 or from L2 to L1, HT is likely to take longer than PE.

### 4.2. Technical effort in post-editing and human translation

In this section, we analyze the technical effort involved in PE and HT by examining the extent of editing. We quantify this effort through the number of insertions and deletions, which are widely recognized indicators of editing activity in translation process research. These measures provide a tangible representation of the modifications made during the translation process. We aim to answer two specific questions: 1) How does the technical effort, quantified as the sum of insertion and deletion counts, differ between HT and PE? and 2) How does the direction of translation influence the editing effort in both PE and HT? We assume that a decrease in the number of deletions and insertions is indicative of reduced technical effort.

To investigate these research questions, we utilized GLMMs with a Poisson distribution. These models incorporate the mode of translation and directionality as predictors, while considering participants as random effects. Our analysis thus provides insights into how these factors shape the extent of editing during the translation process.

**Fig 2:**
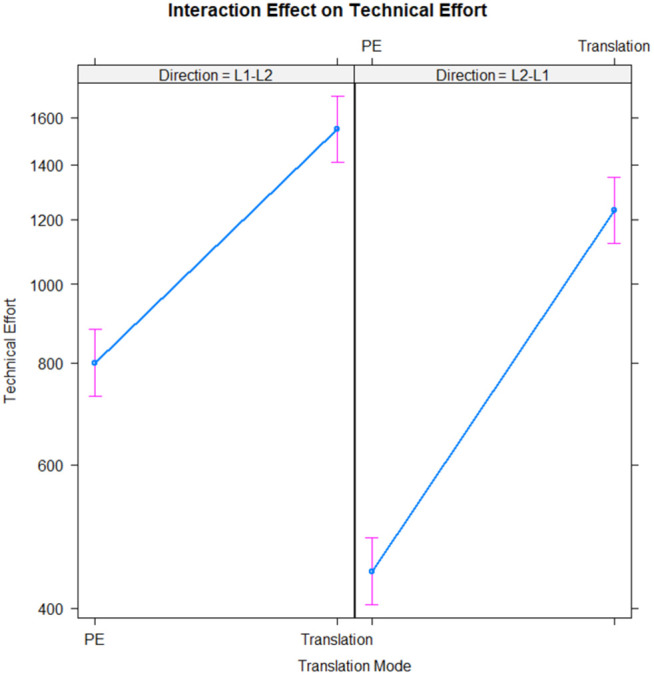
Interaction between translation mode and direction on technical effort.

The model that incorporated the interaction term (Translation Mode * Direction) demonstrated a superior fit and offered a more nuanced understanding of the data, as indicated by an AIC value of 9893.4 and a BIC value of 9907.5. This model revealed significant effects for Translation Mode (Estimate = 0.660089, SE = 0.007665, z = 86.12, p < .001), Direction (Estimate = −0.590006, SE = 0.010421, z = −56.62, p < .001), and the interaction term (Estimate = 0.361544, SE = 0.012400, z = 29.16, p < .001). These results demonstrate that the impact of Translation Mode on Technical Effort is contingent on the Direction. Specifically, translation tasks are more demanding than PE tasks, especially when moving from L1 to L2. The interaction between Translation Mode and Direction on Technical Effort is visualized in Fig 2.

Our insertion analysis exposed a significant interaction between translation mode and direction (b = −0.11, SE = 0.015, z = −7.30, p < .001). Human translations resulted in a higher number of insertions compared to PE (b = 1.24, SE = 0.011, z = 110.01, p < .001), with the L1-L2 direction accounting for more insertions than the L2-L1 direction (b = 0.23, SE = 0.013, z = 17.50, p < .001).

Similarly, our deletion analysis highlighted a meaningful interaction between translation mode and direction (b = −0.35, SE = 0.025, z = −13.85, p < .001). HT had fewer deletions compared to PE (b = 0.12, SE = 0.021, z = 5.54, p < .001), and the L1-L2 direction necessitated more deletions than the L2-L1 direction (b = 1.14, SE = 0.018, z = 64.03, p < .001).

To sum up, our research indicates that technical effort, as measured by insertions and deletions, is significantly influenced by both the mode and the direction of translation. Human translations generally require a greater amount of technical effort compared to PE tasks. Additionally, the direction of translation has a significant impact on the required effort, with translations from L1 to L2 typically demanding more effort than those from L2 to L1. These insights underscore the importance of considering task-specific characteristics and directionality when evaluating the cognitive and technical requirements of the translation process.

### 4.3. Product quality of post-editing and human translation

This section investigates how translation mode (HT vs PE) and directionality (L1-L2 vs L2-L1) impact the quality of the translated product across both language directions. We use quality scores as a measure of translation output, with higher scores indicating superior quality. Our analysis aims to reveal potential differences in output quality between PE and HT, as well as any effects of translation direction on the final product.

A linear mixed-effects model was fitted to examine the effects of translation mode and directionality on product quality scores. The model included random intercepts for participants to account for individual differences in translation performance. The results of the model are summarized in Table 1.

**Table 1:** Fixed effects for product quality scores.

Variable	B	SE	t	p
Intercept	83.59	0.88	95.08	<.001
Mode (Translation)	−5.16	1.18	−4.37	<.001
Direction (zh-en)	−2.86	1.18	−2.42	.018
Mode * Direction (Translation zh-en)	4.55	1.67	2.72	.008

Note. B = Unstandardized regression coefficient; SE = Standard Error; t = t-value; p = significance level.

The quality of the translation was significantly affected by both translation mode and directionality. Specifically, translation tasks were associated with lower quality scores compared to PE tasks (b = −5.16, p < .001). Furthermore, the directionality of the translation from L1-L2 resulted in lower quality scores compared to L2-L1 translations (b = −2.86, p = .018). However, there was a significant interaction effect between translation mode and directionality (b = 4.55, p = .008), indicating that the effect of translation mode on quality scores depends on the directionality.

The variability in quality scores was partially attributed to individual differences in translation performance, as evidenced by the significant random effects for participants (SD = 1.51).

**Fig 3:**
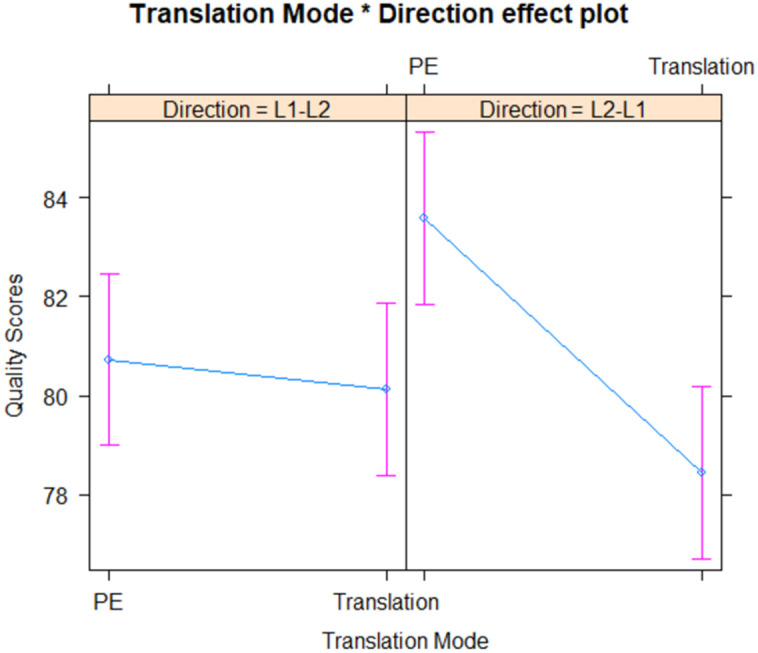
Interaction between translation mode and direction on quality scores.

These results provide evidence for significant differences in the quality of translation outputs based on translation mode and directionality. As visualized in Fig 3, the overall quality scores are influenced by these factors, with translation tasks and L1-L2 direction typically associated with lower scores. However, the interaction effect suggests that the directionality can moderate the impact of translation mode on quality scores.

Overall, these findings highlight the importance of considering both translation mode and directionality when assessing translation quality. The significant effects of these factors on translation quality underscore the differential cognitive and technical demands imposed by different translation tasks and directions.

An independent samples t-test was performed to compare the scores between graduate and undergraduate students. The results revealed no significant difference in scores between the two groups (t(119.37) = 1.4712, p = .1439).

### 4.4. Perceived task difficulty

This section explores how translation mode and directionality influence participants’ subjective perception of task difficulty, as measured by the adapted NASA-TLX scale described in the Methods section.

A linear mixed-effects model was fitted to examine the effects of translation mode and directionality on the perceived difficulty scores. The model included random intercepts for participants to account for individual differences. The results of the model are summarized in Table 2.

**Table 2:** Fixed effects for perceived difficulty scores.

Variable	B	SE	t	p
Intercept	4.23	0.19	22.63	<.001
TaskType (Translation)	0.27	0.22	1.24	.219
Direction (zh-en)	1.06	0.22	4.78	<.001
TaskType * Direction (Translation zh-en)	0.43	0.31	1.38	.171

The perceived difficulty was significantly affected by the translation direction, with L1-L2 translations being perceived as more difficult than L2-L1 translations (b = 1.06, p < .001). However, there was no significant difference in perceived difficulty between HT and PE tasks (b = 0.27, p = .219). The interaction between translation mode and directionality was also not significant (b = 0.43, p = .171), suggesting that the effect of translation mode on perceived difficulty does not depend on directionality.

**Fig 4:**
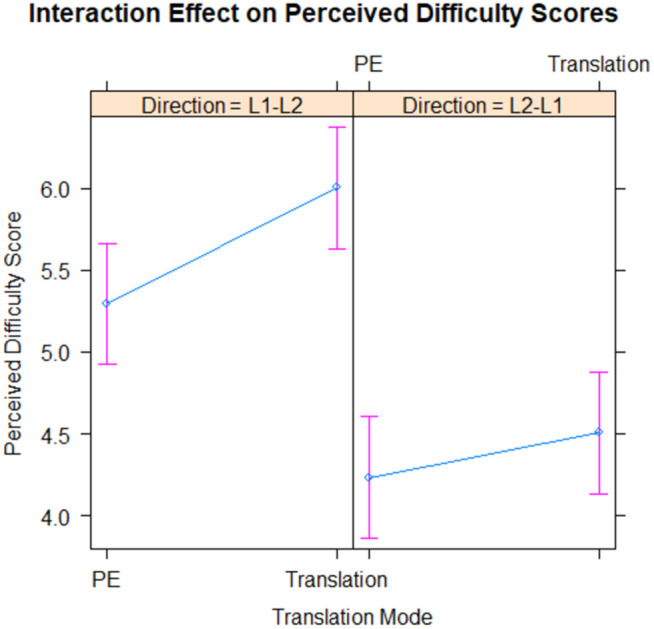
Interaction between translation mode and direction on perceived difficulty scores.

The random effects for participants (SD = 0.57) suggest considerable variation in perceived difficulty scores among participants. Fig 4 shows the effect of translation mode and directionality on perceived difficulty.

These results indicate that directionality significantly affects perceived task difficulty, with L1-L2 translations being perceived as more difficult. However, translation mode does not seem to significantly affect perceived difficulty. Furthermore, the effect of translation mode does not appear to vary with directionality, as indicated by the non-significant interaction effect.

These findings suggest that directionality may have a stronger influence on perceived task difficulty than translation mode. In other words, whether a text is translated from L1 to L2 or vice versa could play a larger role in determining how difficult a translator perceives the task to be than whether they are translating from scratch or PE.

Finally, the significant random effect for participants shows that perceived task difficulty can greatly vary between individuals. This variability highlights the importance of considering individual differences when studying perceived difficulty in translation tasks.

Supplementary analyses revealed no significant differences in perceived difficulty between graduate and undergraduate student translators (t(120.77) = 0.16, p = .875), further underscoring the primacy of directionality over other individual factors like education level.

## 5. Discussion

This section contextualizes our findings within the broader landscape of translation studies, focusing on how our observations contribute to the ongoing discourse on PE and HT processes. We will discuss the implications of our results regarding temporal and technical effort, product quality, and perceived difficulty, rather than restating the findings themselves.

### 5.1. Temporal and technical effort comparison

Our findings on temporal and technical efforts in translation both support and extend the studies discussed in Section 2.1. The observed advantage of PE over HT in terms of speed aligns with Terribile’s [7] large-scale study, which reported PE to be 66% faster on average. However, our results provide specific insights into this trend for the Chinese-English language pair, contributing to a more nuanced understanding of PE efficiency across different language combinations.

The significant effect of directionality on task duration, with L2-L1 translations requiring less time than L1-L2 for both translation modes, corroborates findings by Jia et al. [32] and Da Silva et al. [4]. Their reported time savings of 3.4% to 19% in PE tasks are consistent with our observations, further solidifying the understanding of directionality’s impact on translation speed.

Our analysis of technical effort revealed fewer insertions and more deletions during PE compared to HT, regardless of directionality. This pattern aligns with the findings of Sun et al. [8], who noted that the advantage of PE over HT in terms of technical effort was more pronounced for more educated translators and less technical texts. Our study extends this understanding by demonstrating the consistency of this pattern across both translation directions in the Chinese-English language pair.

These findings reflect the complex relationship between effort and output in PE, as discussed by Cui et al. [9]. While we found PE to be generally less effortful, the extent of this advantage likely varies depending on factors such as text type and translator expertise.

By confirming broader trends in PE efficiency while providing language pair-specific insights, our study contributes to a more comprehensive understanding of the temporal and technical aspects of the translation process. These results lay the groundwork for future investigations into how these dynamics might vary across different text types and levels of translator expertise within the Chinese-English language pair, potentially refining theoretical models of translation processes.

### 5.2. Product quality assessment comparison

Our analysis of product quality scores revealed that PE generally produced higher quality translations than HT, particularly in L2-L1 direction. This finding aligns with the broader trend observed in previous research (e.g., [17]) while providing specific insights for the Chinese-English language pair.

The superior performance of PE in our study supports Garcia’s [18] observations about the advantages of PE. However, our results extend this understanding by demonstrating how these advantages manifest in the Chinese-English context. The particular strength of PE in L2-L1 translations suggests that the combination of machine accuracy and human linguistic expertise is especially effective when translators work into their native language.

Interestingly, our findings on L1-L2 translations partially diverge from previous research. While we observed PE outperforming HT in both directions, the advantage was less pronounced in L1-L2 tasks. This nuanced result adds to the ongoing discussion about the impact of directionality on translation quality, as highlighted by Cui et al. [9] in their study on text types and PE effort.

Our results also contribute to the discourse on the challenges of post-editing NMT output. Unlike Yamada’s [11] findings on the difficulties posed by NMT’s human-like errors, our study suggests that, at least for the Chinese-English pair, translators were able to effectively leverage NMT output to produce high-quality translations. This discrepancy might be attributed to factors such as language pair specifics, translator expertise, or advancements in NMT systems.

Comparing our results with Sun et al.’s [8] study on Chinese-English translation, we found similar trends in PE quality. However, our study provides additional insights into the interaction between translation mode and directionality, offering a more comprehensive picture of these dynamics in the Chinese-English context.

These findings underscore the complex interplay between translation mode, directionality, and output quality. Such investigations could further refine our understanding of when and how PE can most effectively enhance translation quality, potentially informing both theoretical models and practical applications in the field.

### 5.3. Perceived task difficulty comparison

Our findings on perceived task difficulty reveal the enduring impact of directionality, with L1-L2 translations consistently perceived as more challenging, regardless of translation mode. This result both corroborates and extends previous research on the influence of translation direction on perceived difficulty (e.g., [33]). The persistence of this effect across both HT and PE tasks suggests that the cognitive demands of producing text in a non-native language remain substantial, even with machine assistance.

Interestingly, our study found no significant difference in perceived difficulty between HT and PE tasks, contrasting with earlier research that suggested PE might be more challenging due to the need to adhere to pre-translated content [6]. This discrepancy aligns with more recent findings by Jia & Sun [5], who observed that PE was significantly less difficult than HT only when high-quality MT was paired with complex texts. Our results thus contribute to the evolving understanding of PE difficulty perception, possibly reflecting improvements in MT quality over time.

The absence of a significant interaction effect between translation mode and directionality on perceived difficulty in our study adds nuance to the findings of Stasimioti et al. [26]. While they found that inverse PE (L1-L2) required less cognitive effort than direct PE (L2-L1), our results suggest that this difference may not significantly impact overall difficulty perception in the Chinese-English language pair.

Our findings also relate to the observations of Wang & Daghigh [10] regarding the influence of text type on translator effort. While our study did not directly examine text type variations, the substantial variation in perceived difficulty among participants suggests that factors beyond directionality and translation mode play crucial roles in shaping task perception. This aligns with Wang & Daghigh’s emphasis on the significant influence of text type on a translator’s time investment, mental process, and technical input in both HT and PE processes.

The individual variations in perceived difficulty observed in our study underscore the subjective nature of task perception. By confirming the importance of directionality while revealing the complex interplay of factors influencing perceived difficulty, our study contributes to a more nuanced understanding of cognitive demands in translation tasks. These insights offer valuable direction for future research, particularly in exploring how variables such as text type, MT quality, and individual translator characteristics interact to shape task perception in the Chinese-English translation context.

## 6. Conclusion

This study examined the impact of translation mode (HT vs. PE) and directionality (L1-L2 vs. L2-L1) on the translation process, product quality, and translator perception within the Chinese-English language pair. Our findings reveal complex interactions between these factors, contributing valuable insights to the field of translation studies.

PE demonstrated advantages over HT in speed, technical effort, and output quality, particularly in the L2-L1 (English-Chinese) direction. Directionality significantly influenced perceived task difficulty, with L1-L2 translations being perceived as more challenging, while translation mode did not affect perceived difficulty. These results highlight the intricate interplay between translation mode and directionality, underscoring the need for nuanced approaches to translation processes.

Future research should explore interactions between variables such as text types, MT quality levels, and source text complexity with translation mode and directionality. The impact of individual translator characteristics on the translation process and output quality warrants deeper examination. Extending this research to other language pairs and incorporating eye-tracking methodologies could provide more comprehensive insights. Longitudinal studies tracking the evolution of PE practices as NMT systems improve, and utilizing large language models (LLMs) such as ChatGPT and Claude for machine translation in comparative studies, could yield valuable insights for both theoretical models and practical applications.

Limitations of this study include the subjective evaluation of text comparability, small sample size, and lack of eye-tracking data. These constraints suggest opportunities for developing more robust methods for establishing text equivalency across languages, exploring the influence of various text types and genres on translation processes, and integrating eye-tracking technology to capture cognitive effort more comprehensively.

In conclusion, this study advances our understanding of the complex dynamics in translation processes, particularly in the context of PE and HT for the Chinese-English language pair. By addressing the identified limitations and exploring new avenues, future studies can further refine theoretical models and practical applications in the field of translation studies.

## Supporting information

S1 Appendix Experimental materials. (PDF)

S1 Dataset (XLSX)
