# Supplementary material for: Direction matters: Comparing post-editing and human translation effort and quality
Source: PLoS One. 2025 Jul 29;20(7):e0328511. doi: 10.1371/journal.pone.0328511 (PMC12306749; doi:10.1371/journal.pone.0328511)
Supplement: S1 Appendix — (PDF) [file pone.0328511.s001.pdf]

Passage 1:

At its Smart Life showroom in downtown Shenzhen, Huawei Technologies Co displays everything from tablets to vacuum cleaners as it seeks to woo consumers in China's southern tech hub.

But the most eye-catching offering from the Chinese technology giant is a navy blue sport-utility vehicle, the Aito M5, which Huawei launched last week in its latest bid to crack the country's highly competitive electric vehicle (EV) market.

The Aito M5 comes equipped with Huawei's proprietary operating system, HarmonyOS Smart Cockpit, and the vehicle was developed in a partnership with Chinese automotive brand Seres. HarmonyOS currently runs on 220 million smart devices such as phones and watches. Smart Cockpit helps drivers and passengers control vehicle functions such as navigation and music seamlessly, much as they would use a Huawei smartphone.

(129 words)

Source: https://www.scmp.com/tech/big-tech/article/3161760/can-huawei-reinvent-itself-successful-car-brand-its-smartphone

Passage 2:

工信部印发通知部署各地提升 5G 服务质量，明确运营商不得误导、强迫用户办理或升级 5G 套餐，而 4G 套餐查询、办理入口则不得进行隐蔽设置。

通知指出，目前部分电信企业用户提醒不到位、宣传营销不规范等情形引发社会广泛关注。为维护用户权益，推动 5G 持续健康发展，运营商等应提醒用户可通过实体营业厅、客服热线、网上营业厅、手机 APP 查询本地区 5G 网络覆盖情况，在 5G 网络暂未覆盖的地区发展 5G 用户，应事先提醒用户知悉本地区 5G 网络覆盖进度情况。

(205 words)

Source: http://it.people.com.cn/n1/2021/0204/c1009-32022074.html

Machine translation of passage 1:
在其位于深圳市中心的智能生活展厅，华为技术公司展示了从平板电脑到吸尘器的各种产品，以吸引中国南方科技中心的消费者。
但这家中国科技巨头最引人注目的产品是一款深蓝色的运动型多功能车 Aito M5，这是华为上周推出的最新产品，旨在打入中国竞争激烈的电动汽车 (EV) 市场。
Aito M5 配备了华为专有的操作系统 HarmonyOS Smart Cockpit，该车辆是与中国汽车品牌 Seres 合作开发的。
HarmonyOS 目前运行在 2.2 亿台手机、手表等智能设备上。 Smart Cockpit 可帮助驾驶员和乘客无缝控制导航和音乐等车辆功能，就像使用华为智能手机一样。
Source: https://translate.google.com
Accessed on February 28, 2022 21:10

Machine translation of passage 2:
The Ministry of Industry and Information Technology issued a notice to deploy various places to improve the quality of 5G services, clarifying that operators should not mislead or force users to apply for or upgrade 5G packages, and 4G package inquiries and entry points should not be set up in a hidden way.
The notice pointed out that at present, some telecommunications enterprise users have not been reminded in place, and the publicity and marketing have not been standardized, which has caused widespread concern in the society. In order to protect the rights and interests of users and promote the sustainable and healthy development of 5G, operators should remind users to check the coverage of 5G networks in the region through physical business halls, customer service hotlines, online business halls, and mobile APPs, and develop 5G in areas not covered by 5G networks. Users should remind users in advance of the progress of 5G network coverage in the region.
Source: https://translate.google.com/
Accessed on February 28, 2022 21:15
